# The influence of environmental pollution with fluorine compounds on the level of fluoride in soil, feed and eggs of laying hens in Central Pomerania, Poland

**DOI:** 10.1007/s10661-020-8143-3

**Published:** 2020-02-16

**Authors:** Elżbieta Bombik, Antoni Bombik, Katarzyna Rymuza

**Affiliations:** Faculty of Agrobioengineering and Animal Husbandry, Siedlce University of Natural Sciences and Humanities, Prusa Street 14, 08-110 Siedlce, Poland

**Keywords:** Fluorine contamination, Soil type, Plant, Eggshell, Egg contents

## Abstract

The present study was aimed at evaluating fluorine contamination of the eggs of free-ranging laying hens in Northern Poland, in the Central Pomerania region, in relation to the distance from the emission sources. Fluorine levels in the soil, feed, and the shells, and contents of the eggs were assayed with the potentiometric method using an ion-selective electrode from ORION Ion Meter. The sampled eggs were subjected to pressure microwave digestion with the use of a Milestone MLS-1200 microwave. All the samples were digested in 5 ml of supra-pure grade concentrated HNO_3_ from Merck. The mean level of fluorine in the studied soils ranged from 3.79 mg kg^−1^ of DM in typical river alluvial soil to 126.19 mg kg^−1^ of DM in lessive soil. The study revealed an average fluorine content in the feeds administered to the hens on the farms in zone 1 (17.29 mg kg^−1^ of DM), it being 3.5 times higher than the corresponding content in zone 2 (4.92 mg kg^−1^ of DM). A statistically significantly higher mean fluorine level was identified in the eggshells of hens on zone 1 farms, located closer to the pollution emission sources (17.52 mg kg^−1^ of DM), the value being more than 3-fold higher than that in zone 2 (5.47 mg kg^−1^ of DM). The present study revealed an almost twice as high fluorine mean content in the hen eggs collected on farms in zone 1 (1.488 mg kg^−1^ of DM) compared with the hen egg contents in the experimental zone 2 (0.640 mg kg^−1^ of DM), the difference being statistically significant.

## Introduction

Fluorine and fluorides are toxic substances. Their steadily growing presence in the environment requires constant monitoring. Fluorine presence in the soil stems from the use of phosphoric fertilisers which contain slightly more than 1.5% of fluorine. Soils fertilised with superphosphate often accumulate fluorine in the tilth. Contamination of soil with fluorine leached from phosphogypsum stacks is a global environmental issue (Wang et al. 2018). Fluorine can also be found in the surface layer of industrially contaminated soils. The overall fluorine content in the soil largely depends on the type of host rock. Heavier soils tend to contain more fluorine than light soils (Kabata-Pendias and Pendias 1999). Influenced by the growing fluorine contamination of the soil, the content of calcium and magnesium in analysed plants diminishes (Szostek and Ciećko 2017a). Low fluorine levels (ranging from 100 to 200 mg kg^−1^) in the soil have positive effects on the numerical strength of microorganisms and the activity of soil enzymes (Szostek et al. 2015). The filtration of fluorine deep into the profile is predominantly the characteristic of acidic and salty soils. The natural range of fluorine content in Poland is 20–63 mg kg^−1^ in sandy soils, 168–196 mg kg^−1^ in luvisols, 250–323 mg kg^−1^ in clayey soils and 750–1660 mg kg^−1^ in loam soils (Michna 1994; Kabata-Pendias and Pendias 1999).

Fluorine is generally not easily available for plants. However, it can be excessively consumed by plants residing on contaminated soils (Senkondo et al. 2018). Szostek and Ciećko (2017b) showed the effect of soil contamination with fluorine on the yield and chemical composition of fluorine depended on the species and organ of a tested plant. The results of studies suggest a decrease in fluorine levels in the soil and plants in association with a growing distance from the emitter of the element (Franzaring et al. 2006). Studies conducted within the emission range of the chemical plant Zakłady Chemiczne “Police” S.A. did not, however, reveal such clear-cut interdependencies (Kłódka et al. 2008). Positive effects of fluorine on plants have not been identified yet, although fluorine is commonly present in plant tissues. Kowalczyk (1994) found that fluoride-contaminated soil with the pH within the range of 7.0–7.9 does not contribute to increasing fluorine levels in the plant mass. Alkaline and neutral soils with high calcium and magnesium levels typically have a low content of soluble fluorine compounds. Fluorine has a toxic effect on plant respiration and photosynthesis (Kumar et al. 2017). Plant resistance to fluorine toxicity and the limitation of fluorine intake are enhanced by optimal levels of macroelements: N, P, K, Ca and Mg (Kabata-Pendias and Pendias 1999). This is conditioned by low assimilability of soil fluorine compounds (representing mostly insoluble forms) and the protective mechanisms of root systems precluding the absorption, particularly in the case of high pH values in the soil.

Considering the role played by fluorine for human and animal health, it has been determined that fluorine levels in feeds should not exceed 35–40 mg kg^−1^ of DM (usunąć kropkę) (NRC 2001). The admissible level of fluorine, as an undesirable substance in feed compounds, amounts to 150 mg kg^−1^, except for feeds of animal origin in which the level can reach values up to 500 mg kg^−1^. The admissible fluorine level in complete feed mixes for poultry (excluding chicks) and fish is 350 mg kg^−1^, whereas that for chicks is 250 mg kg^−1^ (Regulation of the Minister of Agriculture and Rural Development on the Content of Undesirable Substances in Feeds, dated 6 February 2012, The Official Journal of Laws 2012, it. 203).

Fluorine is one of those microelements which, when received in small quantities, are required for the proper functioning of living organisms. On the other hand, when in excess, it is noxious (Pasternak and Truchliński 1999). Fluorine is regarded as an environmental and industrial contaminant with untoward effects on different organs in humans and animals (Liu et al. 2013). Fluorine has recently been officially recognised as a carcinogenic agent (Machoy-Mokrzyńska 2000). A safe fluorine dose for farm animals should not exceed 1.8 mg per 1 kg of body weight. Animals normally consume a small amount of fluorine in their diet, without negative consequences.

Fluorides can enter the food chain of poultry with industrial contaminants, feed components and potable water (Maurice et al. 2002). High fluorine doses absorbed by laying hens from drinking water are finally bound as fluorides in the eggshell during its mineralisation. The eggshell has been observed to be equipped with a pore system that enables gaseous exchange on the principle of diffusion. Fluoride accumulation in the eggshell does not lead to elevated fluorine content in the egg contents. The shell constitutes a specific filter that purifies the air penetrating the egg. This is important in view of the fitness of eggs from contaminated areas for safe consumption (Machaliński et al. 1996). Górecki et al. (2006) identified the highest fluorine levels in the muscles, livers, bones, and the contents and shells of eggs of hens which received a feed with a 3% phosphogypsum supplement.

The present study was aimed at evaluating fluorine contamination of the eggs of free-ranging laying hens in Northern Poland, in the Central Pomerania region, in relation to the distance from the emission sources. In order to thoroughly analyse the influence of environmental pollution on fluorine levels in the eggs, the analyses were carried out in the trophic system, soil–plant–egg (shell and contents).

## Materials and methods

The analyses were conducted in Northern Poland, in Central Pomerania, in the vicinity of the Vistula, Łeba and Słupia estuaries, at different distances from the emission sources constituted by Rafineria Gdańska (Gdansk Refinery), Gdańskie Zakłady Nawozów Fosforowych (Gdańsk Phosphorus Fertilizer Plant), Elektrociepłownia Wybrzeże (Wybrzeże Combined Heat and Power Plant), Stocznia Gdańska (Gdańsk Shipyard), Keramzyt Gniew (Gniew Expanded Clay Aggregate Plant) and industrial waste dumps, particularly phosphogypsum, slag and ash landfills.

The analyses involved 60 eggs provided by 2-year-old free-ranging multipurpose Karmazyn-laying hens at 6 homesteads (10 eggs randomly selected from each homestead): 3 farms were located in zone 1, near the Vistula estuary, 50 km to the west of the pollution emission sources and 3 homesteads were situated in zone 2, near the estuaries of Łeba and Słupia, between 80 and 100 km to the west of the pollution sources. The hens were obtained from an extensive husbandry system (a coop with a run). The hens were kept outdoors in flocks of 20–35 birds. Their diet in the spring and summer, at the time of the experiment, was chiefly based on wholegrain cereals (barley, wheat and oats), green feed and, periodically, on cooked potatoes. All feed came from its own farms. No mineral and vitamin additives or prophylactic preparations were applied. The average egg yield of these hens was approximately 180 eggs per year, weighing between 57 and 62 g, with the shell hue ranging from light to dark brown. All methods were carried out in accordance with relevant guidelines and regulations on animal welfare (Regulation of the Minister of Agriculture and Rural Development …, The Official Journal of Laws 2010, it. 344). Material for the study was obtained in accordance with the requirements of the National Ethics Committee for Animal Experiments of the European Union (authorisation number 37/2001 and 36/2011).

The soil samples were collected from cultivated fields and pastures owned by the experimental homesteads once, at the culmination of the growing season (June), using a sampling stick, at the depth of 15 cm, into hermetic plastic bags, according to the principle of sample representativity. Each farm provided 6 samples of each soil type, respectively. Dried soil samples were ground in an agate mortar and passed through a 0.25 mm sieve. The pH was assayed for soil samples suspended in a 1 M KCl solution with a potentiometric method. The soil sample was air-dried, and 1 g of the sample was mixed with 10 ml of aqua regia and digested in a Teflon bomb at 60 °C for 90 min. The volume of the digested sample was adjusted to 100 ml with distilled water in a polypropylene volumetric flask. Then, 2 ml of the sample was mixed with 8 ml of 45% sodium acetate solution to make the pH of the final solution neutral. Then, the sample was diluted with TISAB.

The samples of feeds were systematically collected into hermetic plastic bags at each stage of introduction into the bird diet, according to the principle of sample representativity. Each farm provided 6 samples of each feed type, respectively. The plants were cut at 3 cm above the ground level in 3 locations to obtain approximately 1 kg of fresh material. The fodder was washed with distilled water and dried at 800 °C for 24 h. Then, dried material was ground to pass through a 60-mesh sieve. The powered material (2 g) was taken into a 150-ml plastic beaker and 100 ml of 0.1 M perchloric acid was added. The mixture was stirred continuously on a magnetic stirrer for at least 20 min. The suspension obtained was divided into two parts for duplicate analysis.

Whole fridge-stored eggs were kept at 4 °C. Fluorine content measurements in the examined eggs were taken on the 7th day from laying. The eggs were of similar weight (58–60 g) and had no visible shell defects.

The sampled eggs underwent pressure microwave digestion with the use of a Milestone MLS-1200 microwave oven (Rodushkin et al. 1998). All the samples were digested in 5 ml of supra-pure grade concentrated HNO_3_ from Merck.

Fluorine content in the soil, feed, and the shells and contents of the eggs was assayed with the potentiometric method using an ion-selective electrode from ORION (Orion Research 2000), and fluorine levels were presented in milligram/kilogram of DM. The performance of the electrode is affected by several factors, including the pH and temperature of the working solution and the presence of interfering ions in the test solution. The pH of the sample was kept around 5.5. The temperature of standard solutions and samples was equalized and did not differ significantly during calibration and analytical procedures. To eliminate ions containing Fe^3+^, Al^3+^, Ca^2+^, Cu^2+^ and Mg^2+^ which may interact with fluoride, TISAB (buffer for adjusting total ionic strength) was used. Samples and reagents for fluoride analysis were stored in polyethylene containers. Glass containers were avoided because the silica present in the glass may react with fluoride thus giving erroneous results.

The numerical results were profiled using the arithmetic mean ($\overline{\mathrm{x}}$), extreme values (min., max.) and the coefficient of variation (V%).

The results concerning fluorine presence in the experimental soils and feeds were statistically investigated using two-way analysis of variance with interaction, according to the following model:

$$ \mathrm{yijl}=\mathrm{m}+\mathrm{ai}+\mathrm{bj}+\mathrm{abij}+\mathrm{eijl}, $$

where yijl are the values of the investigated parameter for the ith level of factor A (zone) and the jth level of factor B (soil/feed), m is population mean, ai is the effect of the ith level of factor A (zone), bj is the effect of the jth level of factor B (soil/feed), abij is the effect of the interaction between factors A and B, eijl is the random effect.

The results for fluorine levels in the shells and contents of the eggs were analysed using the model of two-way hierarchical classification:

$$ \mathrm{yijl}=\mathrm{m}+\mathrm{ai}+\mathrm{bij}+\mathrm{eijl}, $$

where y_ijl_ are the values of the investigated parameter for the ith level of factor A (zone) and the jth level of factor B (farms within the zones), m is the population mean, a_i_ is the effect of the ith level of factor A (zone), b_ij_ is the effect of the jth level of factor B (farms within the zones), e_ijl_ is the random effect.

The means were compared in detail on the basis of Tukey’s test at the significance level of 0.05.

The methodology for the statistical calculations was taken from Trętowski and Wójcik (1991).

The assessment of fluorine accumulation required the calculation of the contamination rate (ratio of the mean fluorine level in the shell and contents of the eggs in zones 1 and 2) and the bioaccumulation rate (ratio of the mean fluorine level in the shell and contents of the eggs in zones 1 and 2 in relation to the mean fluorine level in the feed). We also calculated the ratio of fluorine levels in the egg shell and contents.

## Results and discussion

The soils at the farms located in zone 1 had pH values (Table 1) ranging from 6.0 in the lowland peat bog soil to 6.3 in the typical river alluvial soil. Slightly higher pH values were identified in the soils at the homesteads situated in zone 2—between 6.2 in the lowland peat bog soil and 6.8 in the typical river alluvial soil. Among the analysed soils, the lowest pH was noted in the lowland peat bog soils and the highest in the typical river alluvial soils both in zones 1 and 2.

**Table 1:** The pH values of the soil types (*n* = 12, number of sample pools)

Soil type	Zone	
	1	2
Lessive soil	6.1	6.5
Typical pararendzina	6.2	6.3
Typical river alluvial soil	6.3	6.8
Podsolic soil	6.2	6.3
Lowland peat bog soil	6.0	6.2

In an analysis of the influence of the organic matter contained in the peat on phosphogypsum fluoride bondings, Kowalczyk (1994) found that the most intensive fluoride bonding occurred in a narrow pH range (4.2–4.5), whereas the present study revealed a slightly acidic reaction in the soils (pH below 7.0).

The mean fluorine levels in the analysed soils (Table 2) were very diverse and statistically significant (*P* ≤ 0.05). The lowest mean fluorine level was found in the typical river alluvial soil (3.79 mg kg^−1^ of DM), and the highest, almost 30-fold higher in comparison with the luvisol, in the lessive soil (126.19 mg kg^−1^ of DM). Similar fluorine levels were identified in these soils both in zones 1 and 2. Statistically significantly higher fluorine content was observed in the zone 1 soils (67.63 mg kg^−1^ of DM) in comparison with the experimental zone 2 soils (60.80 mg kg^−1^ of DM). The content was significantly higher in zone 1 lessive soils (134.67 mg kg^−1^ of DM), typical prarendzinas (88.99 mg kg^−1^ of DM) and podsolic soils (52.03 mg kg^−1^ of DM) as compared with corresponding levels in zone 2. The coefficients of variance in the fluorine levels in the analysed soils were negligible (did not exceed 15.0%), except for the typical river alluvial soil (slightly higher, at 20.1% and 16.9% in zones 1 and 2, respectively).

**Table 2:** Fluorine content in the analysed soils (milligram/kilogram of DM)

Soil type	Basic Parameters	Zone		Mean
		1	2	
Lessive soil	Arithmetic mean $\Big(\overline{\mathrm{x}}$)	134.67^e^	117.71^e^	126.19^e^
	Extreme values (min.–max.)	112.00–157.00	106.00–126.00	
	Coefficient of variation (V%)	7.6	4.8	
Typical pararendzina	Arithmetic mean $\Big(\overline{\mathrm{x}}$)	88.99^d^	79.78^d^	84.38^d^
	Extreme values (min.–max.)	79.00–101.40	69.10–92.00	
	Coefficient of variation (V%)	7.2	6.9	
Typical river alluvial soil	Arithmetic mean $\Big(\overline{\mathrm{x}}$)	4.25^a^	3.32^a^	3.79^a^
	Extreme values (min.–max.)	2.99–6.02	2.75–4.87	
	Coefficient of variation (V%)	20.1	16.9	
Podsolic soil	Arithmetic mean $\Big(\overline{\mathrm{x}}$)	52.03^b^	46.50^b^	49.27^b^
	Extreme values (min.–max.)	44.69–60.02	40.05–52.04	
	Coefficient of variation (V%)	7.2	7.4	
Lowland peat bog soil	Arithmetic mean $\Big(\overline{\mathrm{x}}$)	58.20^c^	56.70^c^	57.45^c^
	Extreme values (min.–max.)	50.00–63.00	51.4–61.1	
	Coefficient of variation (V%)	4.7	4.3	
Mean		67.63^b^	60.80^a^	64.22
LSD_0.05_ for the types of soil 2.78				
LSD_0.05_ for the zones 1.26				
LSD_0.05_ for the interaction soil types x zones 3.94; 2.83				

^a, b, c, d, e^The means in the columns (except for the zone means) marked with the same letters do not differ significantly (*P* ≤ 0.05)

The mean fluorine level in the analysed soils did not exceed the range of natural fluorine content in these types of soil (Kabata-Pendias and Pendias 1999). Michna (1994) reported that the soil fluorine balance largely depends on the amount of loamy minerals and the soil pH. The most intensive sorption of this element in the soil occurs in periods of extremely acidic or alkaline reaction. Even in the case of only slightly acidic pH, the present study revealed statistically significantly higher fluorine levels in the lessive soils in comparison with the other soils both in experimental zones 1 and 2. The study by Sarinana-Ruiz et al. (2017) shows the overall fluorine concentration in the surface fluorine-contaminated soil layer ranging from 89.75 to 926.63 mg kg^−1^ of DM, with the fluorine-specific value exceeded in many places (321 mg kg^−1^ of DM). The fluorine content in the soils investigated by Drzymała et al. (1995) ranged from 63 to 399 mg kg^−1^ of DM and depended on the direction of pollutant migration. According to Jha et al. (2008), the mean total fluoride concentration in surface soil near the field of brick in the northern periphery of the Lucknow, one of the oldest urban agglomeration in India, varied from 322 to 456 μg g^−1^. The maximum levels of F were observed in samples of shallow aquifers close to agricultural and metallurgical areas, which implies the frequent use of fertilisers and the impact of industrial wastewater in the industrial area of Pakistan (Qurat-ul-Ain et al. 2017). All of the soil sites after the closure of Anglesey Aluminium Metals Ltd. smelter in Great Britain had a mean fluoride concentration 199− 328 mg F kg^−1^ (Brougham et al. 2013).

The analysis of the mean fluorine content in the feeds (Table 3) administered to the laying hens revealed statistically significant differences between most feeds (except for the levels in oat and barley grain). The highest mean fluorine level was found in potatoes (24.07 mg kg^−1^ of DM). It was more than 14 times higher than the mean fluorine level in wheat grain (1.65 mg kg^−1^ of DM), with greater differentiation between the feeds in zone 1 (more than 18 times) in relation to zone 2 (slightly more than 8 times). The study revealed a mean fluorine content in the feeds administered to the hens on the farms in zone 1 (17.29 mg kg^−1^ of DM) that was 3.5 times higher than the corresponding content in zone 2 (4.92 mg kg^−1^ of DM). The difference was statistically significant. Statistically confirmed differences were identified in the fluorine levels between the zones for almost all the feeds, except for the content in wheat grain (2.08 and 1.21 mg kg^−1^ of DM in the particular zones). The coefficient of variance for the fluorine content in the feeds varied considerably, ranging from slightly more than 11.0 to over 35.0%. The highest value of the coefficient of variance for the fluorine content was identified in barley grain (19.9 and 35.1% in the zones, respectively).

**Table 3:** Fluorine content in the analysed feeds (milligram/kilogram of DM)

Type of feed	Basic Parameters	Zone		Mean
		1	2	
Green feed from pastures	Arithmetic mean $\Big(\overline{\mathrm{x}}$)	5.20^b^	3.23^ab^	4.21^b^
	Extreme values (min.–max.)	3.78–6.12	2.28–4.44	
	Coefficient of variation (V%)	11.5	16.7	
Wheat grain	Arithmetic mean $\Big(\overline{\mathrm{x}}$)	2.08^a^	1.21^a^	1.65^a^
	Extreme values (min.–max.)	1.29–2.72	1.02–1.67	
	Coefficient of variation (V%)	15.4	12.0	
Oat grain	Arithmetic mean $\Big(\overline{\mathrm{x}}$)	22.48^d^	4.47^bc^	13.47^c^
	Extreme values (min.–max.)	16.29–30.07	2.19–7.03	
	Coefficient of variation (V%)	18.9	26.7	
Barley grain	Arithmetic mean $\Big(\overline{\mathrm{x}}$)	18.52^c^	5.76^c^	12.14^c^
	Extreme values (min.–max.)	11.44–25.61	3.65–11.03	
	Coefficient of variation (V%)	19.9	35.1	
Potatoes	Arithmetic mean $\Big(\overline{\mathrm{x}}$)	38.19 ^e^	9.95 ^d^	24.07 ^d^
	Extreme values (min.–max.)	28.90–48.50	6.80–22.30	
	Coefficient of variation (V%)	12.2	30.5	
Mean		17.29^b^	4.92^a^	11.11
LSD_0.05_ for the types of feed 1.46				
LSD _0.05_ for the zones 0.66				
LSD _0.05_ for the interaction feed types x zones 2.07; 1.49				

^a, b, c, d, e^As explained in Table 2

It should be stressed that the hens in zone 1 received feeds with physiologically excessive fluorine levels for the plants, ranging from 1.0 to 1.6 mg per 100 g of DM (Machoy et al. 1988). Excessive mean fluorine levels were determined in oat and barley grain and cooked potatoes. This goes against the claim that plants in polluted areas predominantly absorb fluorine compounds from the air (through their stomas and epiderm), while the intake of the compounds through the root systems from the soil is minimal, and water-soluble fluorides chiefly accumulate in the leaves (Pasternak and Truchliński 1999; Gautam et al. 2010). Similar ranges of fluorine content in green feed were observed by Zakrzewska and Orowicz (1997). Research Gautam et al. (2010) carried out in Nagaur district (India) showed that fluoride concentration in cereal crops ranged from 1.88 to 15.88 μg g^−1^. In a study of Yadav et al. (2012) conducted in Dausa district in India, the concentration of fluoride in the wheat yield ranged between 3.24 and 14.3 μg g^−1^. While in potatoes, it was at the level of 1.22–2.92 μg g^−1^. In this study, the concentration of fluoride in potatoes was estimated at 1.22 to 2.92 μg g^−1^, and these levels were almost 10 times lower than in the case of own studies. Fluoride concentrations of maize grains were greater in the proximity of brick kilns than in more distant sites and was 154.6 mg kg^−1^(dw) (Ahmad et al. 2014). The concentration of fluoride in vegetation at polluted sites after the closure of Anglesey Aluminium Metals Ltd. smelter in Great Britain had decreased to background levels of 10 mg F kg^−1^ after 36 weeks (Brougham et al. 2013). According to Ahmad et al. (2012), visible injury symptoms, hydrogen fluoride concentrations in air and foliar fluoride concentrations were all greater in the vicinity of brick kilns in Asia compared with more distant sites, indicating that fluoride emissions from brick kilns were the main cause of damage. Considering the role played by fluorine for human and animal health, it was determined that fluorine levels in feeds should not exceed 35–40 mg kg^−1^ of DM. (NRC 2001). The mean fluorine content in the potatoes administered to the hens in zone 1 was within this range. According to Wahid et al. (2014), F content in mango leaves and fruits of all varieties grown near a brick kiln field in southern Pakistan was significantly higher compared with their counterparts at the control site, located 20 km from brick ovens. The F content in the leaves was 4–6 times greater than the corresponding values for fruits. Saleem et al. (2015) recommend that farmers in the vicinity of the ceramic industry and brick kilns in Pakistan, where there may be higher concentrations of fluoride in the soil and in the atmosphere, use plant varieties less sensitive to these pollutants. Yadav et al. (2012) recommend the cultivation of plants with relatively low fluorine enrichment capabilities, such as those with seeds or tubers as the main edible parts, in areas contaminated with fluoride.

The experiment showed that fluorine compound concentration in hen eggshells (Table 4) increased considerably along with the growing exposure of the birds. What is striking is the dispersion of the results, from 3.02 mg kg^−1^ of DM in the eggshells of hens at a homestead in zone 2 to 33.85 mg kg^−1^ of DM on a zone 1 farm. A statistically significantly higher mean fluorine level was identified in the eggshells of hens at zone 1 homesteads, located closer to the pollution emission sources (17.52 mg kg^−1^ of DM), the value being more than 3-fold higher than that in zone 2 (5.47 mg kg^−1^ of DM). Statistically significant differences in the mean fluorine content in the eggshells were observed between the farms. The highest significant fluorine level was found in the eggshells of hens kept on farm 2 (22.90 mg kg^−1^ of DM), while the lowest value was identified in the eggshells of laying hens on farm 1 (13.02 mg kg^−1^ of DM). The differences between the eggshell fluorine levels in zone 2 proved insignificant and oscillated between 4.31 and 7.73 mg kg^−1^ of DM. The dispersion of this parameter, measured with the magnitude of the coefficient of variance, was quite extensive and exceeded 15.0% (except for farm 4 in zone 2).

**Table 4:** Fluorine levels in the hen eggshells (milligram/kilogram of DM)

Zone	Farm	Basic parameters			Mean
		Arithmetic mean $\Big(\overline{\mathrm{x}}$)	Extreme values (min.–max.)	Coefficient of variance (V%)	
	1	13.02^a^	10.41–17.32	16.4	
1	2	22.90^c^	15.33–33.85	29.6	17.52^b^
	3	16.65^b^	12.36–21.32	18.4	
	4	4.38^a^	3.11–5.02	14.2	
2	5	4.31^a^	3.02–5.56	19.6	5.47^a^
	6	7.73^a^	5.93–11.12	24.2	
Overall mean					11.50
LSD_0.05_ for the zones 1.70					
LSD_0.05_ for the farms within the zones 3.54					

^a, b, c^The means in the columns marked with the same letters do not significantly differ (*P* ≤ 0.05)

Similarly, increased fluorine levels in the eggshells were determined on farms located in the vicinity of a phosphogypsum dump near Gdańsk (Juszkiewicz and Szkoda 1986). Uziębło et al. (1993) observed the eggshell fluorine content to decrease with the rising distance from the farm to the emission source of environmental pollution. Miao et al. (2017) found fluorine concentration in the yolk and eggshell of laying hens to be positively correlated with fluorine levels in the hen diet. They also found that calcium and phosphorus metabolism disorders, caused by growing fluorine content in the diet, affect the formation of the eggshell by diminishing its strength and thickness.

The present study revealed that the fluorine mean in the contents of the hen eggs (Table 5) collected on farms in zone 1 (1.488 mg kg^−1^ of DM) was almost twice as high as in the hen egg contents in the experimental zone 2 (0.640 mg kg^−1^ of DM), the difference being statistically significant. The findings also revealed statistically significant differences between fluorine levels in the contents of hen eggs at all the homesteads located in zone 1 (the highest on farm 3, at 1.725 mg kg^−1^ of DM, and the lowest on farm 1, at 1.155 mg kg^−1^ of DM) and between farm 6 (0.720 mg kg^−1^ of DM) and farms 4 and 5 (0.610 and 0.590 mg kg^−1^ of DM, respectively) in zone 2. The extreme fluorine content values in the egg contents in zone 1 exceeded the threshold of 1.000, ranging from 1.002 to 1.945 mg kg^−1^ of DM. They were, in turn, much lower in zone 2, between 0.528 and 0.852 mg kg^−1^ of DM. This parameter varied little both in zones 1 and 2, predominantly remaining under 10.0% (except for one farm in zone 1). The dispersion was much lower compared with the variance of the fluorine content in the eggshell, particularly on farm 2 in the first zone (5-fold lower, 5.8% in relation to 29.6%).

**Table 5:** Fluorine levels in the hen egg contents (mg·kg^−1^ of DM)

Zone	Farm	Basic parameters			Mean
		Arithmetic mean $\Big(\overline{\mathrm{x}}$)	Extreme values (min.–max.)	Coefficient of variance (V%)	
	1	1.155 ^a^	1.002–1.345	10,2	
1	2	1.585 ^b^	1.492–1.785	5,8	1.488 ^b^
	3	1.725 ^c^	1.622–1.945	5,3	
	4	0.610 ^a^	0.528–0.703	7,1	
2	5	0.590 ^a^	0.533–0.666	6,8	0.640 ^a^
	6	0.720 ^b^	0.622–0.852	9,8	
Overall mean					1.064
LSD_0.05_ for the zones 0.042					
LSD_0.05_ for the farms within the zones 0.087					

^a, b, c^As explained in Table 4

In comparison with the present study, lower fluorine amounts in the egg contents, ranging from 0.2 to 0.4 mg kg^−1^ of DM, were identified by Waldbott (1962). On the other hand, Ensminger (1995) found fluorine levels inside the eggs at an average of 1.3 mg kg^−1^ of DM. The physiologically correct fluorine content remains within the range of 0.5–1.3 mg kg^−1^ of DM for the egg inner substance (Jamroz 1996; Sulikowska 1996) and between 1 and 90 mg kg^−1^of DM for the shell. (Górecki 1998; European Council Directive 1999) depending on the concentration of fluorine on land and in water in the area. Assuming these values as adequate, we can see the physiological standard for the mean fluorine level is exceeded in the contents of hen eggs from zone 1 (especially on farms 2 and 3).

The high level of fluorine accumulation (Table 6) is corroborated by the shell and contents contamination rates of the hen eggs, much above 1.000 (3.203 and 2.325, respectively), with fluorine content higher in zone 1 than in zone 2. The eggshell bioaccumulation rates, both in zones 1 and 2, exceeded the 1.000 level (at 1.013 and 1.112, respectively), which is indicative of higher fluorine content in the shell in comparison with the feed. On the other hand, the bioaccumulation rates were lower in the egg contents (0.086 and 0.130 for the particular zones). The eggshell fluorine content in experimental zone 1 was almost 12-fold higher (11.774) in comparison with the level of this element in the egg contents, whereas this proportion was lower (8.346) in zone 2. It should be pointed out that, in comparison with the shell, the contamination and bioaccumulation rates in the egg contents were lower. However, the bioaccumulation indices for both the shells and contents of the (more contaminated) zone 1 eggs were slightly lower as compared with zone 2. The range of variability of fluorine levels in the shell-to-contents ratio on the farms was more than twice as large in zone 1 (14.448) as compared with zone 2 (7.180).

**Table 6:** Degree of fluorine accumulation in the shells and contents of hen eggs

Accumulation parameters	Strefa—zone		Mean
	1	2	
Contamination rate			
Eggshell			3.203
Egg contents			2.325
Bioaccumulation rate			
Eggshell	1.013	1.112	1.035
Egg contents	0.086	0.130	0.959
Fluorine level ratio			
Between the egg shell and contents (S/C*)	11.774	8.346	10.808
(Scale of variance at the homesteads)	(9.652–14.448)	(7.180–10.736)	

*S/C fluorine content ratio of the egg shell and substance

Górecki et al. (2006) observed the highest fluorine levels in the egg substances and shells of hens which received a feed with 3% phosphogypsum supplementation. The authors found the mean value of the S/C index increased from 19.6 to 41.3 along following increased phosphogypsum supplementation in the laying hen diet. According to Górecki et al. (2006), excessive fluorine amounts consumed by hens are evacuated by the shells, and possibly with faeces, which prevents fluorine overaccumulation in the edible parts of eggs. This is also indicative of complex interactions between fluorine and other elements present in the shell and contents of eggs (Lifshitz 1997).

## Conclusions

The analyses revealed that fluorine levels rise in the soil, feed and the contents and shells of hen eggs with diminishing distance from the source of emission of fluorine compounds. The mean level of fluorine in the soils under analysis did not exceed the natural ranges for the soil fluorine content. A 3.5-fold higher mean fluorine level was identified in the mixes fed to the hens in zone 1 (greater pollution). In the case of oat and barley grain and cooked potatoes, the obtained values exceeded the physiological standards for the plants. The concentration of fluorine compounds in the shells and contents of the eggs considerably increased in line with the growing exposure of the laying hens. Despite the protective fluorine-accumulating function of the eggshell, the egg contents of zone 1 hens were observed to have exceeded physiological fluorine levels. Compared with the shell, the contamination and bioaccumulation rates in the egg contents were lower. However, the bioaccumulation indices of both the shell and contents of zone 1 eggs were slightly lower as compared with zone 2. The eggshell fluorine level in experimental zone 1 was almost 12-fold higher than the level of this element in the egg contents, whereas this proportion was lower in zone 2.
